# High‐efficiency genome editing by Cas12a ribonucleoprotein complex in *Euglena gracilis*

**DOI:** 10.1111/1751-7915.14393

**Published:** 2024-02-08

**Authors:** Toshihisa Nomura, June‐Silk Kim, Marumi Ishikawa, Kengo Suzuki, Keiichi Mochida

**Affiliations:** ^1^ RIKEN Center for Sustainable Resource Science Yokohama Japan; ^2^ RIKEN Baton Zone Program Yokohama Japan; ^3^ Faculty of Agriculture Yamagata University Tsuruoka Japan; ^4^ Institute of Plant Science and Resources Okayama University Okayama Japan; ^5^ Euglena Co., Ltd. Tokyo Japan; ^6^ Kihara Institute for Biological Research Yokohama City University Yokohama Kanagawa Japan; ^7^ Graduate School of Nanobioscience Yokohama City University Yokohama Kanagawa Japan; ^8^ School of Information and Data Sciences Nagasaki University Nagasaki Japan

## Abstract

Transgene‐free genome editing based on clustered regularly interspaced short palindromic repeats (CRISPR) technology is key to achieving genetic engineering in microalgae for basic research and industrial applications. *Euglena gracilis*, a unicellular phytoflagellate microalga, is a promising biomaterial for foods, feeds, cosmetics and biofuels. However, methods for the genetic manipulation of *E. gracilis* are still limited. Here, we developed a high‐efficiency, transgene‐free genome editing method for *E. gracilis* using *Lachnospiraceae bacterium* CRISPR‐associated protein 12a (LbCas12a) ribonucleoprotein (RNP) complex, which complements the previously established Cas9 RNP‐based method. Through the direct delivery of LbCas12a‐containing RNPs, our method reached mutagenesis rates of approximately 77.2–94.5% at two different *E. gracilis* target genes, *Glucan synthase‐like 2* (*EgGSL2*) and a phytoene synthase gene (*EgcrtB*). Moreover, in addition to targeted mutagenesis, we demonstrated efficient knock‐in and base editing at the target site using LbCas12a‐based RNPs with a single‐stranded DNA donor template in *E. gracilis.* This study extends the genetic engineering capabilities of *Euglena* to accelerate its basic use for research and engineering for bioproduction.

## Graphical abstract

We developed a genome editing method for *Euglena gracilis* using LbCas12a RNP complex. We achieved mutagenesis rates of 77.2–94.5% at two *E. gracilis* target genes. We also demonstrated targeted knock‐in and base editing in *E. gracilis.*

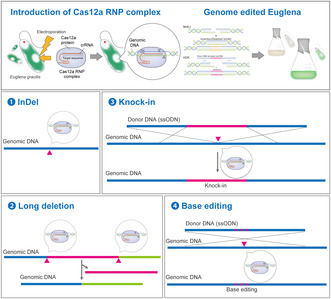

## INTRODUCTION

Clustered regularly interspaced short palindromic repeats (CRISPR)–based genome editing technology has versatile genetic applications in functional genomics analysis, gene therapy and molecular breeding (Doudna & Charpentier, 2014; Zhang, 2019). Two widely used genome editing tools based on the Class II CRISPR system include the CRISPR‐associated proteins Cas9 and Cas12a (also named Cpf1 [CRISPR of *Prevotella* and *Francisella*]), which are classified as type II and type V, respectively. Currently, CRISPR/Cas9‐based genome editing is the most widely used genome editing technology in various species (Doudna & Charpentier, 2014; Zhang, 2019). The popular nuclease *Streptococcus pyogenes* Cas9 (SpCas9) targets 20‐bp sequences, recognizes NGG as its protospacer adjacent motif (PAM) and induces a blunt‐ended DNA double‐strand break (DSB) in the target sequence 3 bp upstream of the PAM (Doudna & Charpentier, 2014; Zhang, 2019).

Conversely, the RNA‐guided endonuclease Cas12a has different features in the editing machinery compared to Cas9‐based editing. First, Cas12a requires a T‐rich PAM sequence for target site recognition (Bandyopadhyay et al., 2020; Khan & Sallard, 2023; Manghwar et al., 2019; Zetsche et al., 2015). Additionally, Cas12a generates a 5‐bp staggered double‐stranded break 18–23 bp downstream of the PAM (Bandyopadhyay et al., 2020; Khan & Sallard, 2023; Manghwar et al., 2019; Zetsche et al., 2015). Hence, Cas12a‐based genome editing facilitates the introduction of long deletions through the repeated DNA cleavage of its target sequence. Second, Cas12a forms a complex with 41–44 nt of CRISPR RNA (crRNA) and does not require an additional trans‐activated CRISPR RNA (tracrRNA) (Zetsche et al., 2015). Target sequences in the Cas12a system are longer than those in the Cas9 system, thus, resulting in greater target specificity and reduced off‐target effects (Kim et al., 2017; Kleinstiver et al., 2016). Thus, Cas12a‐based genome editing approaches have several advantages over the Cas9 system, which can be exploited to further expand the genome editing toolbox in target species. Currently, three Cas12a nucleases are widely used: AsCas12a from *Acidaminococcus* sp., FnCas12a from *Francisella novicida* and LbCas12a from *Lachnospiraceae bacterium* (Bandyopadhyay et al., 2020; Khan & Sallard, 2023). LbCas12a, FnCas12a, Mb2Cas12a (from *Moraxella bovoculi*) and temperature‐tolerant AsCas12a or LbCas12a variants that can maintain their activity even at lower temperatures (<28°C) are suitable for applications in plant and microalgal species (Endo et al., 2016; Ferenczi et al., 2017; Gaillochet et al., 2023; Kleinstiver et al., 2019; Li et al., 2019; Schindele & Puchta, 2020; Wang et al., 2017; Zhang et al., 2021; Zhong et al., 2018).

Genome editing through the direct delivery of ribonucleoprotein (RNP) complexes offers advantages compared with transgene‐based methods, such as reduced off‐target effects, low cytotoxicity, less labour and high efficiency (Wang et al., 2016). In RNP‐mediated genome editing without transgenes, the delivered RNP causes random insertions or deletions (InDels) at the target site via DSBs, which are repaired via non‐homologous end joining (Puchta & Fauser, 2014). Organisms whose genomes are edited using transgene‐free methods may bypass current regulations for genetically modified organisms (GMOs) (Kim & Kim, 2016; Pennisi, 2016). Therefore, RNP‐based genome editing facilitates food and healthcare applications, such as crop breeding and microalgae engineering. However, RNP‐based genome editing in microalgae using selection‐free methods has low mutagenesis efficiency (Baek et al., 2016; Ferenczi et al., 2017; Jeong et al., 2023; Shin et al., 2016; Yoshimitsu et al., 2018); therefore, transgene‐free RNP‐based genome editing technology in microalgae must be further improved to facilitate its practical use.

*Euglena gracilis* is a unicellular photosynthetic flagellated microalga that has recently been used industrially for multiple purposes. *E. gracilis* can accumulate approximately 60–70% of its dry weight of paramylon, which is a crystalline form of β‐1,3‐glucan, as intracellular granules (Harada et al., 2020). In addition to potential bioactive functions of paramylon, such as an immunomodulator or a dietary fibre (Nakashima et al., 2018), its chemical properties enable the use of paramylon as a bioresource for the production of various processed products, such as films, thermoplastics and nanofibers (Feuzing et al., 2022). Moreover, accumulated paramylon is degraded and converted to wax esters (myristyl myristate, C14:0–C14:0) under anaerobic conditions (Inui et al., 2017). Wax esters from *E. gracilis* are a suitable source of bio‐jet fuel because of its low freezing point compared to that of lipids from other microalgal species (Harada et al., 2020). Therefore, *E. gracilis* is a promising bioresource in the establishment of a more sustainable society (Harada et al., 2020). With the aim of enabling genetic modifications to improve the productivity in *E. gracilis*, we previously established a highly efficient genome editing method by direct delivery of Cas9 RNP complexes (Nomura et al., 2019, 2020). However, further expansion of the genetic toolbox is required to achieve a more flexible modification of the genetic code of *E. gracilis*.

Therefore, in this study, to expand the genetic toolbox for *Euglena*, we developed a high‐efficiency genome editing system by direct delivery of LbCas12a RNP complexes. At two target genes, *glucan synthase‐like 2* (*EgGSL2*) and phytoene synthase gene (*EgcrtB*), our method achieved mutagenesis rates of approximately 77.2–94.5%. Moreover, we demonstrated targeted knock‐in and base editing in *E. gracilis* using LbCas12a RNP complexes with single‐stranded oligodeoxynucleotides (ssODNs), thereby providing a base‐editing framework in *Euglena*.

## EXPERIMENTAL PROCEDURES

### Strain and culture conditions

*Euglena gracilis* strain Z was provided by the Institute of Applied Microbiology (Tokyo, Japan) culture collection. It was cultured in Koren–Hutner (KH) medium (pH 5.5 or 3.5) (Koren, 1967) on a rotary shaker (120 rpm) at 28°C under 50 μmol m^−2^ s^−1^ continuous fluorescent light.

### Direct delivery of LbCas12a RNP complexes by electroporation

crRNA was synthesized by Integrated DNA Technologies (Integrated DNA Technologies, IA, USA), and a 100 μM solution was prepared with nuclease‐free duplex buffer (Integrated DNA Technologies). For the preparation of RNP complexes, mixtures were prepared with the crRNA and Alt‐R L.b. Cas12a (Cpf1) Nuclease Ultra (Integrated DNA Technologies, solution at 10 μg μL^−1^) at a ratio of 1:1 (v/v) for 15 min at 20–25°C. Electroporation solution was prepared by mixing Cramer–Myers (CM) medium (pH 5.5) without sodium citrate (Nomura et al., 2020) and 0.3 M sucrose (filter‐sterilized) at a ratio of 2:3 (v/v). *E. gracilis* was cultured in KH medium for 3 days, and 1 mL of culture was centrifuged at 400 × *g* for 30 s. *E. gracilis* cell pellets were washed once with electroporation solution and resuspended with electroporation solution at a cell density of 1 × 10^6^ cells mL^−1^. For electroporation, 0.8 μL of LbCas12a–RNP complex solution was added to 24.2 μL of *E. gracilis* suspension. The mixed solution of RNP complexes and *E. gracilis* suspension was added to a 1‐mm gap cuvette EC‐001 (NEPAGENE, Chiba, Japan). A NEPA21 Super Electroporator (NEPAGENE) was used for the introduction of RNP complexes into *E. gracilis*. Detailed electroporation conditions are described in our previous report (Nomura et al., 2020). One millilitre of KH medium was immediately added after electroporation. Then, the solution was transferred to a 12‐well plate and cultured on a rotary shaker (120 rpm) at 28°C under dark conditions for 96 h for recovery culture. The isolation of single cell‐derived pure strains using the Micro Pick and Place System (NEPAGENE) following the methods described in our previous report (Nomura et al., 2020).

### Microscopy observation

Microscopy images of *E. gracilis* were acquired using a DP27 camera (Olympus, Tokyo, Japan) attached to a CKX53 inverted microscope (Olympus). The frequencies of phenotypically altered cells were manually counted with the cell counter plugin in ImageJ software (Abràmoff et al., 2004) using digital images taken at random. More than 1500 cells were counted in each treatment to determine the percentage of cells with altered phenotypes.

### T7 endonuclease I assay

For the detection of mutagenesis at target sites in each Cas12a RNP‐introduced *E. gracilis*, a T7 Endonuclease I (T7EI) assay was performed using an Alt‐R Genome Editing Detection Kit (Integrated DNA Technologies). Genomic DNA template from *E. gracilis* was extracted using a Kaneka Easy DNA Extraction Kit version 2 (KANEKA, Tokyo, Japan). DNA fragments including the *EgGSL2* target sites were amplified with Tks Gflex DNA Polymerase (Takara Bio, Shiga, Japan) using the *EgGSL2* Check F‐R primer set (Table S1). T7EI treatment was carried out according to the manufacturer's protocol using the amplified DNA fragments. Digested DNA fragments were analysed by agarose gel electrophoresis.

### Amplicon sequencing analysis

Genomic DNA from *E. gracilis* was extracted using a Monarch Genomic DNA Purification Kit (New England Biolabs, MA, USA). PCR products including each target site were amplified with ExTaq DNA polymerase HS (Takara Bio) using each amp‐seq F‐R primer set (Table S1). DNA libraries were prepared by two‐step tailed PCR using the 2nd F‐R primer set (Table S1) and purified by AMPure XP (Beckman Coulter, CA, USA). Paired‐end sequencing (2 × 300 bp) was performed using a MiSeq system (Illumina, CA, USA) with MiSeq Reagent Kit v3 (Illumina). InDel rates, nucleotide distribution, and allele frequency table for each amplicon were analysed using CRISPresso2 software with the following analysis parameters (default_min_aln_score: 60, plot_window_size: 30, quantification_window_center: 0, quantification_window_size: 20, ignore_substitutions: True) (Clement et al., 2019).

### Detection of target mutations by Sanger sequencing

DNA fragments including the *EgGSL2* and *EgcrtB* target sites were amplified with Tks Gflex DNA Polymerase (Takara Bio) using each primer set (Table S1). A CloneJET PCR Cloning Kit (Thermo Fisher Scientific, MA, USA) was used to clone the PCR products. Each resulting plasmid harbouring an insert was purified using a Plasmid DNA Extraction Mini Kit (FAVORGEN, Ping‐Tung, Taiwan). Mutations were detected from Sanger sequencing data.

### Quantification of paramylon content

Paramylon content from approximately 5 mg of dry *E. gracilis* samples was measured by following a previously reported procedure (Muramatsu et al., 2020).

### Growth curve test

For growth curve tests of wild‐type and genome‐edited strains, 150 mL of cell culture medium adjusted to a cell concentration of approximately 1 × 10^5^ cells mL^−1^ using KH medium were shaking cultured at 28°C under 50 μmol m^−2^ s^−1^ continuous fluorescent condition. The cell concentration in the cell culture was measured every 24 h using a cell counter CDA‐1000 (Sysmex, Hyogo, Japan).

### Quantification of chlorophyll *a + b* content

Weighed frozen cell pellet samples of *E. gracilis* were mixed in 1 mL of N,N‐dimethylformamide (DMF) and kept at 25°C for 30 min. The absorbance of the extracted supernatant was measured at 647, 664 and 750 nm using a NanoDrop One C spectrophotometer (Thermo Fisher Scientific). Chlorophyll *a + b* content was calculated using the following equation: chlorophyll *a + b* = 17.67 × (A647–A750) + 7.12 × (A664–A750) (Porra et al., 1989).

### ssODN‐mediated knock‐in and base editing

*EgGSL2*‐targeted ssODNs (Table S1) were synthesized by Ultramer DNA Oligo synthesis service (Integrated DNA Technologies), and a 200 μM solution was prepared with nuclease‐free duplexbuffer (Integrated DNA Technologies). For electroporation, 0.8 µL of RNP complex solutionand 0.5 µL of 200 μM ssODN stock solution were added to 23.2 µL of an *E. gracilis* cell suspension. Knock‐in or base‐editing events were detectedby *Eco*RI‐HF or *Hin*dlll‐HF (New England Biolabs) digestion for 2 h at 37°C of DNA fragments including targetsites that had been amplified by Tks Gflex DNA Polymerase using the *EgGSL2* F‐R primer set (Table S1) and by Sanger sequencing.

## RESULTS

### High‐efficiency transgene‐free targeted mutagenesis using LbCas12a ribonucleoprotein complexes

**FIGURE 1:**
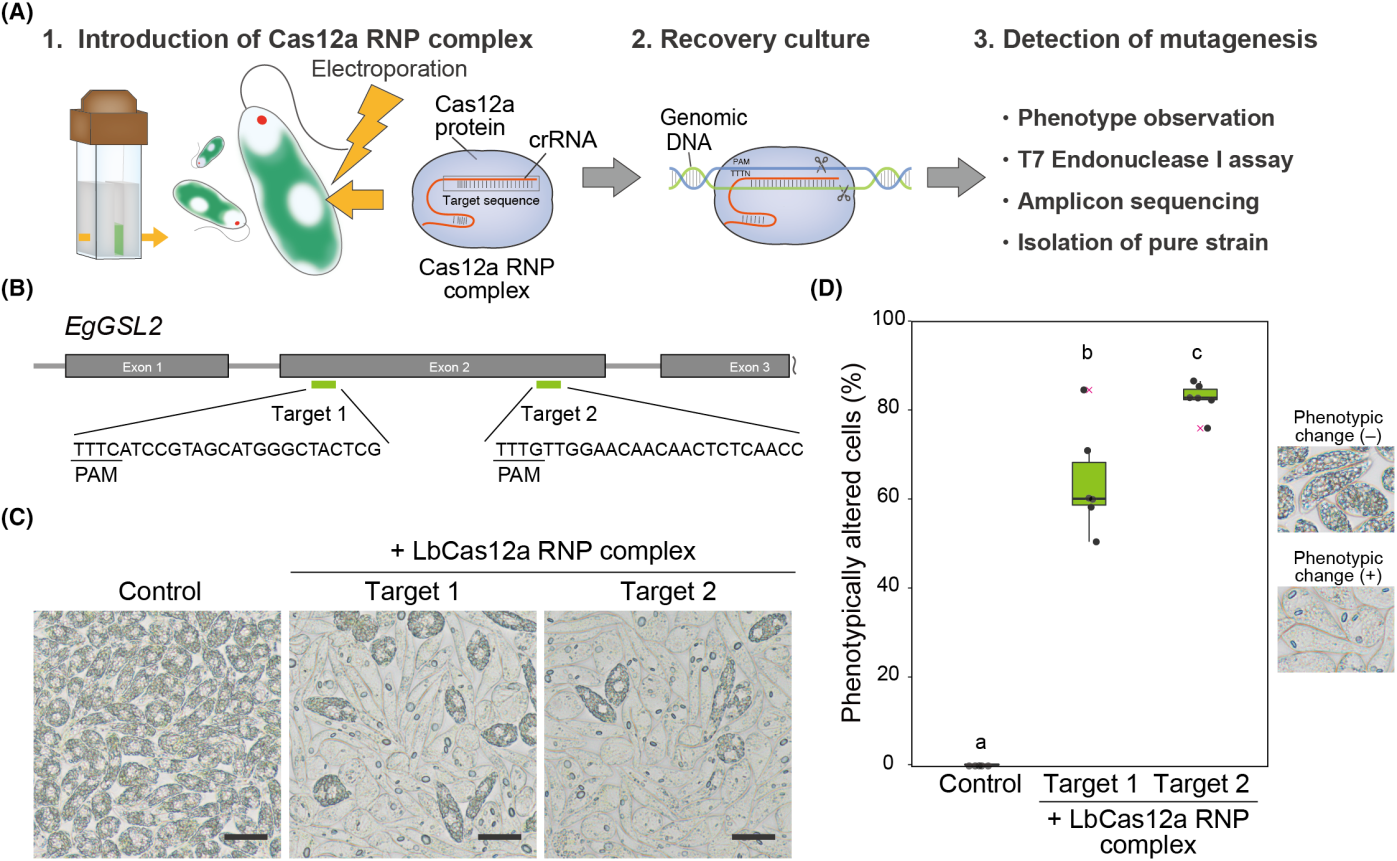
Genome editing of *Euglena gracilis* using LbCas12a RNPs. (A) Overview of experimental procedures for the genome editing of *E. gracilis* using LbCas12a RNP complexes. (B) Design of target sequences 1 and 2 on the 5′ genomic region of *EgGSL2* exon 2. (C) Representative images of cell populations at 96 h after introduction of LbCas12a RNP complexes non‐treated condition (Control) and LbCas12a RNPs targeting *EgGSL2*. Scale bar, 30 μm. (D) Percentage of phenotypically altered cells 96 h after introduction of LbCas12a RNP complexes in the non‐treated condition (Control) and LbCas12a RNP complexes targeting *EgGSL2*. Results of five independent biological replicates counting more than 1500 cells per treatment are shown as a box‐and‐whisker plot. Statistical significance was determined by ANOVA, followed by Tukey–Kramer multiple comparison test. Different letters indicate means that statistically significant differences at *p* < 0.01.

**FIGURE 2:**
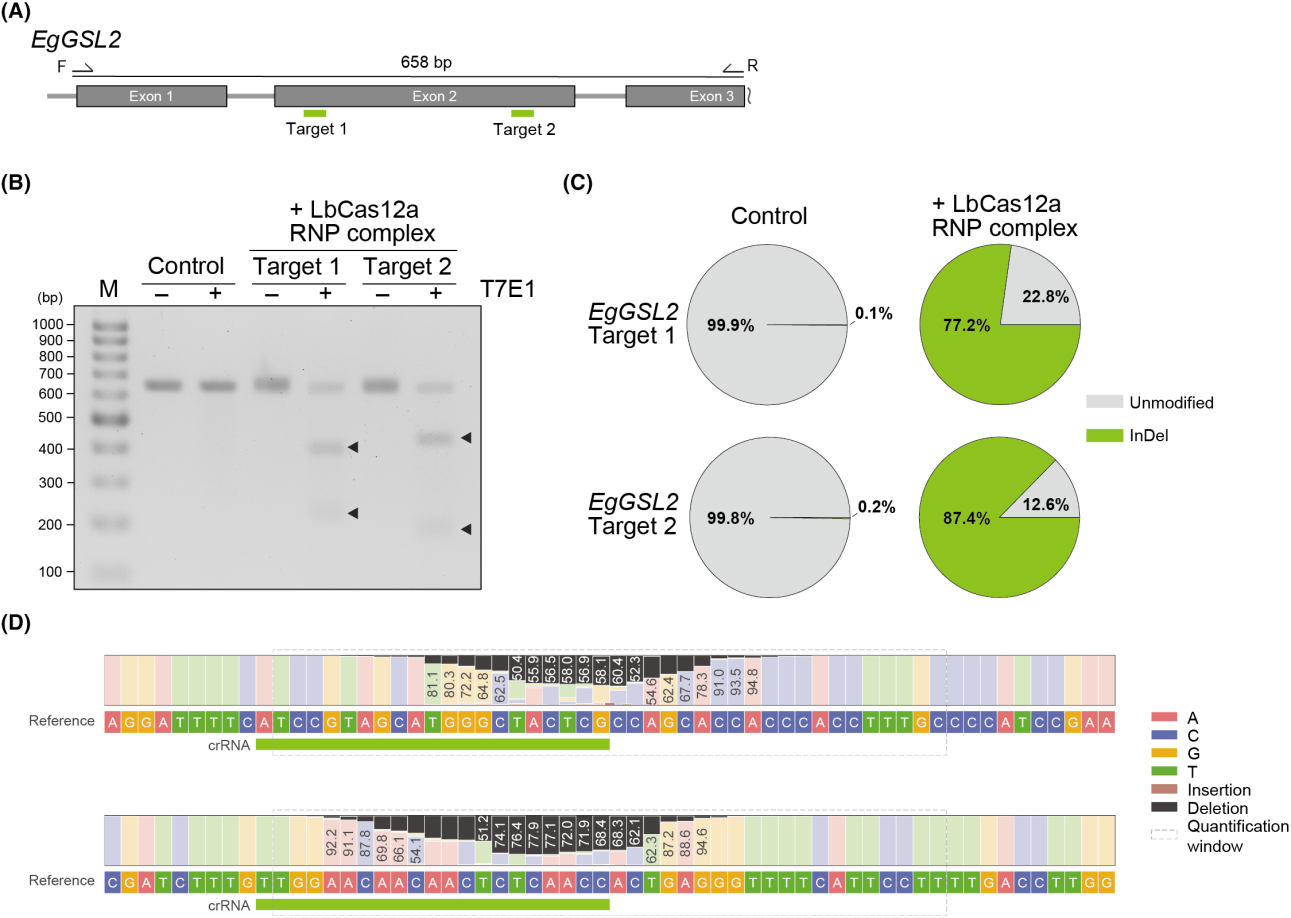
Efficiency of genome editing with LbCas12a RNP complexes. (A) Schematic illustration of the *EgGSL2* gene model and the PCR product containing target sequences 1 and 2. (B) T7 Endonuclease I assay at 96 h after introduction of LbCas12a RNP complexes in the non‐treated condition (Control) and *EgGSL2*‐targeting LbCas12a RNP complexes. Arrowheads indicate the digested PCR products. M, DNA ladder marker. (C) Analysis of the InDel mutation rate at 96 h after introduction of LbCas12a RNP complexes in the non‐treated condition (Control) and *EgGSL2*‐targeting LbCas12a RNPs by amplicon sequencing. Unmodified indicates wild type or substitution. (D) Nucleotide distribution of the amplicon flanking the target sites at 96 h after introduction of LbCas12a RNP complexes in the non‐treated condition (Control) and *EgGSL2*‐targeting LbCas12a RNP complexes analysed with CRISPresso2. Black bars indicate the percentage of reads with bases deleted at that position. Brown bars between the bases indicate the percentage of reads that have an insertion at that position.

To examine genome editing using LbCas12a RNP complexes in *E. gracilis*, we targeted the *EgGSL2* gene (accession number: LC225615). *EgGSL2* encodes an enzyme with paramylon synthase activity, and its knockdown or knockout inhibits the accumulation of paramylon, leading to changes in the morphology of paramylon granules (Nomura et al., 2019; Tanaka et al., 2017). We designed two crRNAs targeting different sites in the second exon of *EgGSL2* and prepared LbCas12a RNP complexes (Figure 1A,B). Then, we introduced the LbCas12a RNP complexes into *E. gracilis* cells by electroporation. We cultured the transfected cells in heterotrophic medium under dark conditions for 96 h to promote recovery and phenotypic emergence (Figure 1A). *E. gracilis* cells transfected with these two *EgGSL2*‐targeted LbCas12a RNP complexes exhibited markedly fewer paramylon granules (Figure 1C). The average frequencies of altered phenotypes for *EgGSL2* targets 1 and 2 were 64.0% and 82.6%, respectively (Figure 1D). To validate mutagenesis events, we assessed the presence of mutation at the *EgGSL2* locus in the cell population transfected with LbCas12a RNP complexes by a T7 endonuclease I (T7EI) assay (Figure 2A,B).

**FIGURE 3:**
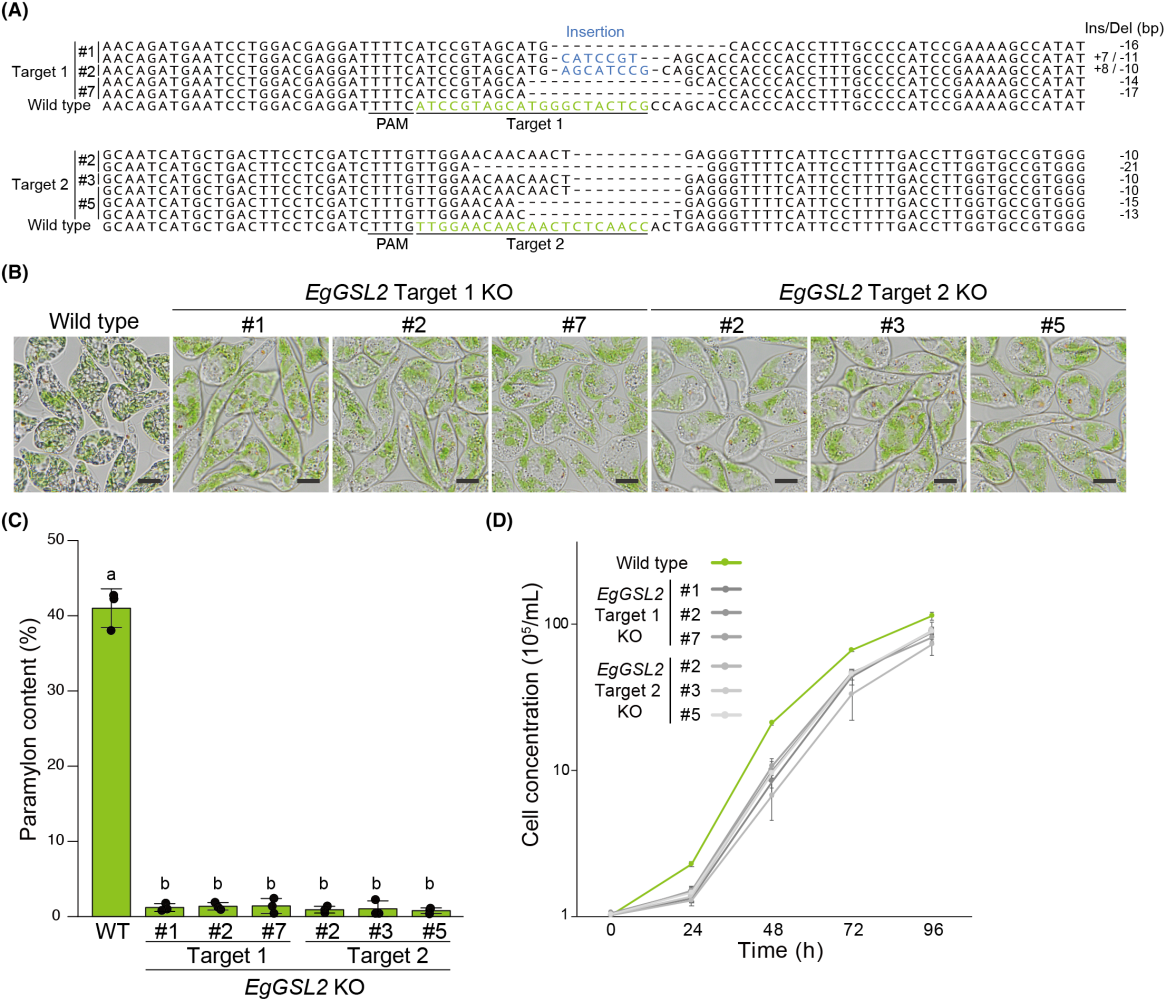
Characteristics of the isolated *EgGSL2* mutant strains established by LbCas12a RNP‐based genome editing. (A) Alignment of genomic DNA sequences flanking the target sites in wild‐type and isolated *EgGSL2* genome‐edited strains. (B) Representative images of the wild‐type and isolated *EgGSL2* genome‐edited strains after 2 days of growth in KH medium. Scale bar, 10 μm. (C) Paramylon content in wild‐type and isolated *EgGSL2* genome‐edited strains. Data are means of three independent biological replicates (*n* = 3) ± SD. Statistical significance was determined by ANOVA, followed by Tukey–Kramer multiple comparison test. Different letters indicate means that statistically significant differences at *p* < 0.01. (D) Growth curves of wild type and isolated *EgGSL2* genome‐edited strains in KH medium culture. Data are means of three independent biological replicates (*n* = 3) ± SD.

To analyse genome editing efficiency using LbCas12a, we performed amplicon sequencing of the *EgGSL2* target regions using population of cells in recovery culture after electroporation. We detected InDel mutation rates at *EgGSL2* targets 1 and 2 of 77.2% and 87.4%, respectively (Figure 2C). By contrast, we measured a significantly lower percentage of InDels in the non‐treated LbCas12a RNP (Control) sample with 0.1 to 0.2% (Figure 2C), the presence of which we attribute to sequencing errors. The most frequent deletion lengths at the target 1 and target 2 sites were 17 bp (13.0%) and 10 bp (20.8%), respectively (Figure 2D, Figures S1A,B and S2A,B). We obtained and analysed a pure genome‐edited strain derived from a single cell with a mutation in the target site of the *EgGSL2* gene. We detected up to three patterns of deletion mutations in each isolated *eggsl2* mutant (Figure 3A) suggesting the presence of a gene family or polyploidy in *E. gracilis* (see Discussion below). *eggsl2* mutant strains obtained by LbCas12a‐based genome editing showed a low paramylon phenotype (Figure 3B), drastically reduced paramylon content and phenotype of cell growth suppression (Figure 3C,D).

We also simultaneously introduced two LbCas12a RNP complexes targeting different sites of *EgGSL2* (target 1 and target 2) and identified mutations that deleted approximately 200 bp of the region between the target sites (Figure S4A–E), indicating that this method can be used to introduce long deletions in a target genomic region. Based on these findings, we demonstrate that our method with LbCas12a RNP complexes enables high‐efficiency, transgene‐free targeted mutagenesis in *E. gracilis*.

### Versatility of transgene‐free targeted mutagenesis using LbCas12a RNP complexes

**FIGURE 4:**
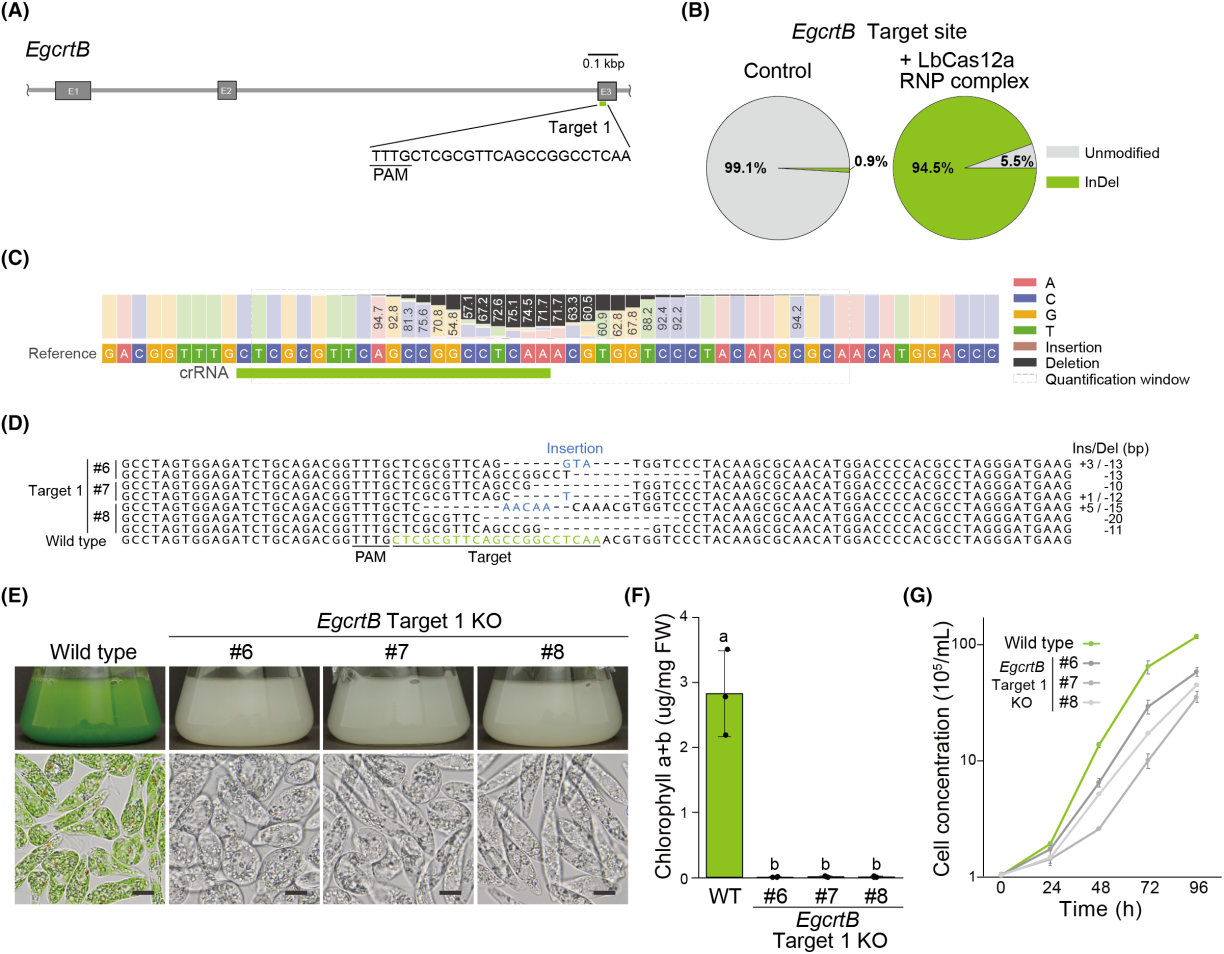
LbCas12a RNP‐based genome editing of *EgcrtB*. (A) Design of the target sequence on the 5′ genomic region of *EgcrtB*. (B) Analysis of the InDel mutation rate at 96 h after introduction of LbCas12a RNP complexes in the non‐treated condition (Control) and *EgcrtB*‐targeting LbCas12a RNP complexes by amplicon sequencing. Unmodified indicates wild type or substitution. (C) Nucleotide distribution of the amplicon flanking the target site at 96 h after introduction of LbCas12a RNP complexes in the non‐treated condition (Control) and *EgcrtB*‐targeting LbCas12a RNP complexes analysed with CRISPresso2. Black bars indicate the percentage of reads with bases deleted at that position. Brown bars between the bases indicate the percentage of reads that have an insertion at that position. (D) Alignment of genomic DNA sequences flanking the target sites in wild‐type and isolated *EgcrtB* genome‐edited strains. (E) Representative images of wild‐type and isolated *EgcrtB* genome‐edited strains after 4 days of growth in KH medium. Upper: Images of cultured liquid. Lower: Microscopy images. Scale bar, 10 μm. (F) Chlorophyll *a + b* content in wild‐type and isolated *EgcrtB* genome‐edited strains. Data are means of three independent biological replicates (*n* = 3) ± SD. Statistical significance was determined by ANOVA, followed by Tukey–Kramer multiple comparison test. Different letters indicate means that statistically significant differences at *p* < 0.01. (G) Growth curves of wild type and isolated *EgcrtB* genome‐edited strains in KH medium culture. Data are means of three independent biological replicates (*n* = 3) ± SD.

To test the versatility of our system, we targeted the *EgcrtB* gene (GenBank accession number LC062707) by LbCas12a RNP‐mediated mutagenesis (Figure 4A). The *EgcrtB* gene encodes phytoene synthase, which is involved in carotenoid and chlorophyll accumulation. Accordingly, its dysfunction causes chlorosis in *E. gracilis* (Kato et al., 2020; Tamaki et al., 2023). We quantified InDel mutations at target 1 of the *EgcrtB* locus by amplicon sequencing using population of cells in recovery culture after electroporation and obtained an average InDel mutagenesis rate of 94.5% (Figure 4B), with a 12‐bp deletion being the most frequent mutation (19.5%) (Figure 4C, Figure S3A,B). As with *EgGSL2*, we isolated a pure strain with a mutation in the target site of *EgcrtB* as three independent mutants (Figure 4D). We observed that *egcrtb* mutant strains exhibit a chlorosis phenotype when cultured under light conditions (Figure 4E), and their chlorophyll *a + b* content was drastically lower than the wild‐type control (Figure 4F). The strains showing the completely chlorotic phenotype were obtained in 14.8–30.8% of the randomly isolated strains from cell culture pool samples after LbCas12a RNP introduction (Table S2). The mutant exhibited a slow cell proliferation phenotype compared to the wild strain under light‐conditioned culture (Figure 4G).

Together with the results of our *EgGSL2* target experiment, our findings suggest that LbCas12a RNP‐mediated genome editing can be used for stable mutagenesis of target genes in *E. gracilis*.

### Targeted knock‐in and base editing using LbCas12a RNP complexes with ssODNs

**FIGURE 5:**
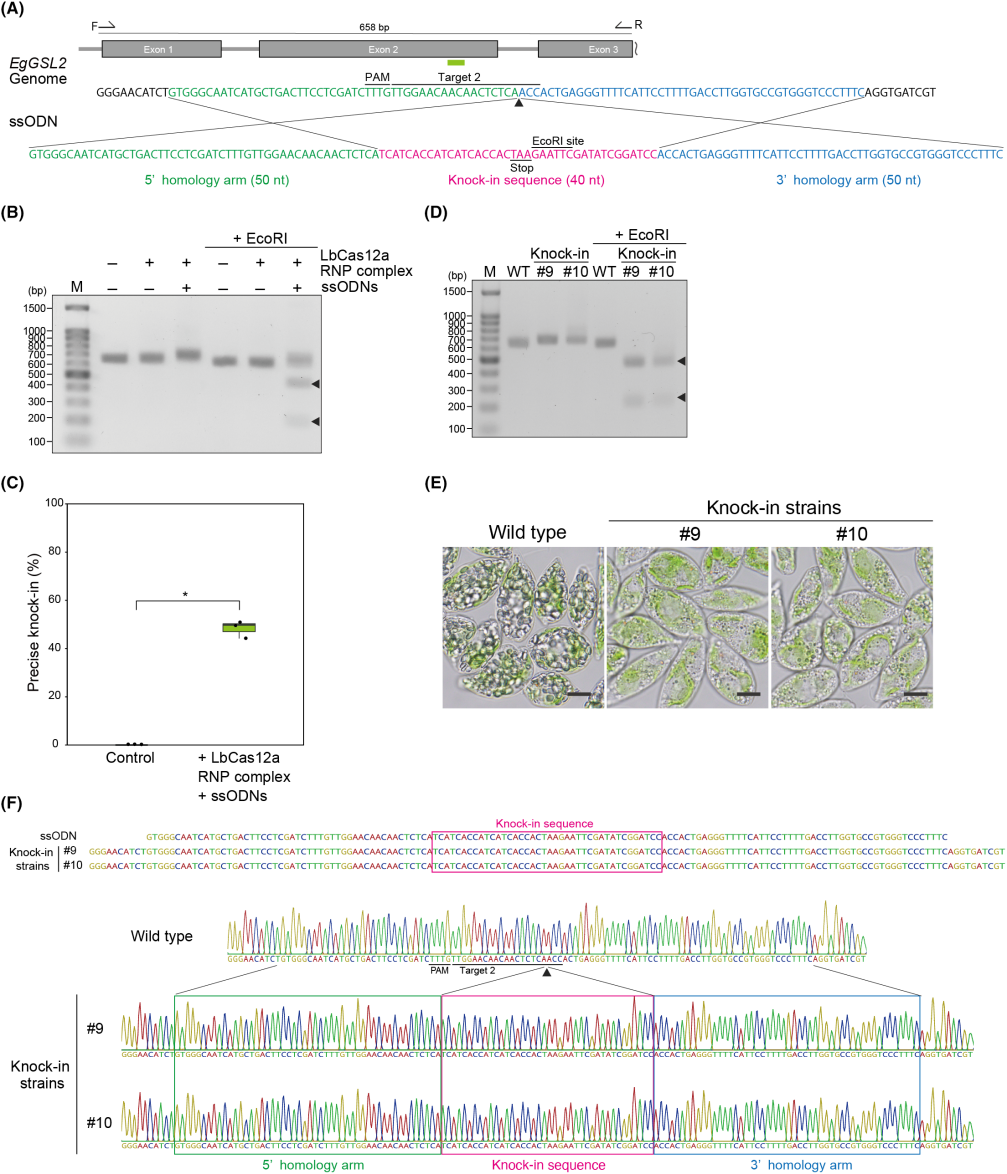
Knock‐in at *EgGSL2* target site using LbCas12a RNP complexes and ssODNs. (A) Design of the ssODN sequence for knock‐in at the *EgGSL2* target site 2. (B) PCR products derived from the cell population 96 h after introduction of LbCas12a RNP complexes in the non‐treated condition (Control) and ssODNs for knock‐in and LbCas12a RNP complexes targeting *EgGSL2*. Arrowheads indicate the *Eco*RI‐digested PCR products. (C) Percentage of precise knock‐in at 96 h after introduction of LbCas12a RNP complexes in the non‐treated condition (Control), *EgGSL2*‐targeting LbCas12a RNPs and ssODNs by amplicon sequencing are shown as a box‐and‐whisker plot. Statistical significance was determined by Welch's t‐test. Asterisks indicate the statistically significant differences at *p* < 0.05. (D) PCR products derived from the wild‐type and isolated *EgGSL2* knock‐in‐type genome‐edited strains. Arrowheads indicate the *Eco*RI‐digested PCR products. (E) Representative images of wild‐type and isolated *EgGSL2* knock‐in‐type genome‐edited strains after 2 days of growth in KH medium. Scale bar, 10 μm. (F) Sanger sequencing peak data of wild‐type and isolated *EgGSL2*  knock‐in‐type genome‐edited strains.

**FIGURE 6:**
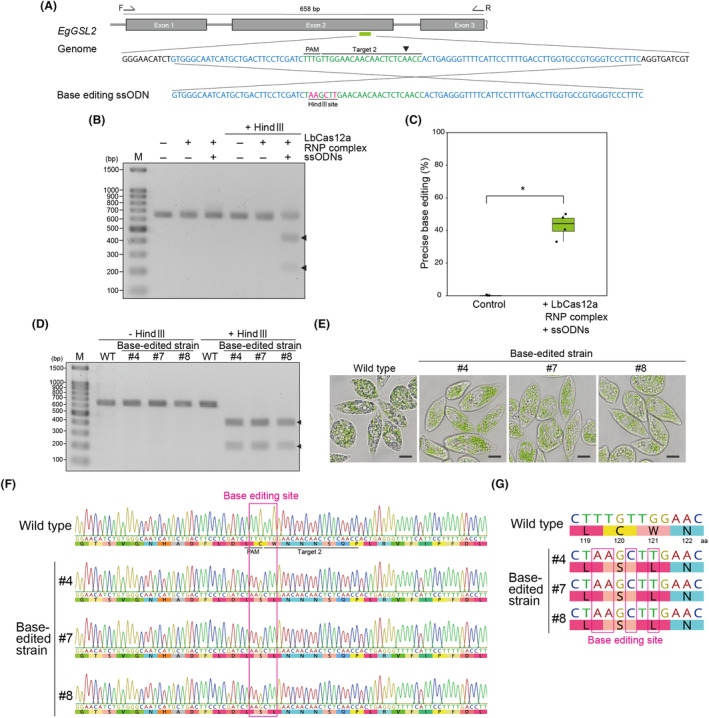
Base editing at a *EgGSL2* target site using LbCas12a RNPs and ssODNs. (A) Design of the ssODN sequence for base editing of the *EgGSL2* target site 2. (B) PCR products derived from the cell population 96 h after introduction of LbCas12a RNP complexes in the non‐treated condition (Control) and ssODNs for base editing and LbCas12a RNP complexes targeting *EgGSL2*. Arrowheads indicate the *Hin*dIII‐digested PCR products. (C) Percentage of precise base‐editing at 96 h after introduction of LbCas12a RNP complexes in the non‐treated condition (Control), *EgGSL2*‐targeting LbCas12a RNPs and ssODNs by amplicon sequencing are shown as a box‐and‐whisker plot. Statistical significance was determined by Welch's *t*‐test. Asterisks indicate the statistically significant differences at *p* < 0.05. (D) PCR products derived from the wild‐type and isolated *EgGSL2* target site base‐edited strains. Arrowheads indicate the *Hin*dIII‐digested PCR products. (E) Representative images of wild‐type and isolated *EgGSL2* target site base‐edited strains after 2 days of growth in KH medium. Scale bar, 10 μm. (F) Sanger sequencing peak data of wild‐type and isolated *EgGSL2* target site base‐edited strains. (G) Alignment of target sites in wild type and base‐edited strains.

To advance LbCas12a RNP‐based genome editing in *E. gracilis*, we performed a precise single‐stranded oligodeoxynucleotide (ssODN)‐mediated knock‐in experiment. We used *EgGSL2* target 2 and LbCas12a RNP complexes containing ssODNs, consisting of 50‐nt sequences upstream and downstream of the *EgGSL2* target 2 site as 5′ and 3′ homology arms that sandwiched a 40‐bp knock‐in DNA fragment containing an *Eco*RI site (Figure 5A). As a result, we detected effective knock‐in events in the ssODN‐treated *E. gracilis* recovering cultured pool and isolated pure strains by restriction digests with *Eco*RI (Figure 5B,D,E). The results of amplicon sequencing analysis of the recovered cell culture pool samples showed that 44.2–50.8% of the amplicon sequences had precise knock‐ins (Figure 5C). We also confirmed the presence of precise knock‐in sequences at the target site through Sanger sequencing of isolated pure strains (Figure 5F). Among the randomly isolated pure strains, 25–43.5% of the strains were identified as having precise knock‐ins (Table S3). Moreover, we induced base editing at the *EgGSL2* target 2 site using an ssODN that introduces a *Hin*dIII recognition site and changes amino acids C121 and W122 to S121 and L122 (Figure 6A). We confirmed that co‐transfection with LbCas12a RNP complexes enables precise base editing at the target sequence (Figure 6B–D,F). According to amplicon sequencing analysis of recovered cell culture pool samples, the percentage of precise base‐edited sequences was 33.3–49.3% (Figure 6C). In randomly isolated strains, base‐edited strains were obtained at 29.6–56.5% (Table S4). We observed that the base‐edited strains with the C121S and W122L amino acid residue conversions in the EgGSL2 protein (Figure 6F,G) show a low paramylon phenotype (Figure 6E), suggesting that these amino acids are important for GSL2 function as a paramylon synthase. Taken together, our results show that LbCas12a–ssODN‐mediated knock‐in and base editing can be applied to E. gracilis and highlight how site‐directed mutagenesis can facilitate the elucidation of enzymatic function in *E. gracilis* at single‐base resolution.

## DISCUSSION

In this study, we established a high‐efficiency Cas12a RNP‐based genome editing method for *E. gracilis*. We assessed its genome editing efficiency by targeting the *EgGSL2* and *EgcrtB* genes and performing amplicon sequencing analysis. We determined that its editing efficiency was 77.2–94.5% (Figures 1 and 2), indicating that it is comparable to the highly efficient Cas9 RNP‐based method we previously established (Nomura et al., 2019). In case of mutations introduced by genome editing negatively affecting cell proliferation, it is possible that the efficiency of mutation introduction assessed after the recovery culture post‐electroporation may have been underestimated. Indeed, the knockout of *EgGSL2* and *EgcrtB* exhibited a reduced proliferation rate in the growth tests (Figures 3D and 4G). However, amplicon sequencing of the cell population four days after the recovery culture detected sufficiently high InDel rate (Figures 2C and 4B). Thus, we considered that the proliferation during the recovery culture did not significantly affect our estimation of the mutation efficiency in the cell population because of such growth bias. Taken together, utilization of the LbCas12a RNP‐based genome editing technique in conjunction with Cas9 RNP‐based genome editing will allow us to expand the selection of target sequences for genome editing in *E. gracilis*.

It is beneficial to widen the selection of target sequences for knock‐in experiments, such as the addition of tags to the 5′ and 3′ termini of target genes and for base‐editing experiments in which amino acid substitutions are generated in specific protein domains because the sites that can currently be targeted by mutagenesis are severely limited due to strict design rules. In this study, we carried out successful knock‐in and base‐editing experiments using LbCas12a RNP complexes and ssODNs (Figures 5 and 6), which will further improve the flexibility of genetic modification techniques in *E. gracilis*. We found that the use of ssODNs resulted in 36.6–54.1% lower efficiency of mutation introduction compared to that with random mutagenesis on *EgGSL2* targets 2 when ssODNs were not used (Figures 2, 5 and 6). On the other hand, employing ssODNs allows for the precise introduction of mutations, such as assured frameshifts and amino acid substitutions, through precise base editing. Therefore, we believe that the method should be chosen according to the specific objectives of the experiment.

The characteristics of the Cas12a PAM enable this nuclease to edit AT‐rich genomic regions, targets that are normally difficult to edit with the Cas9 system. Furthermore, Cas12a‐based genome editing has been reported to have fewer off‐targets (Bernabe‐Orts et al., 2019; Kim et al., 2016; Tang et al., 2018), which may make it suitable for industrial applications. On the other hand, it is difficult to estimate the potential off‐target effects in the crRNA design process because genomic information on *E. gracilis* is still incomplete. Therefore, our current strategy to avoid misleading from phenotypic changes derived from off‐target effects is to confirm the reproducible phenotype observed even with multiple crRNAs designed at different sites within a target gene. We detected a long deletion of more than 10 bp by LbCas12a RNP‐based genome editing in *E. gracilis* (Figures S1–S3), consistent with the previously reported features of Cas12a‐based genome editing (Kim et al., 2017; Li et al., 2019). This characteristic suggests that Cas12a‐based genome editing is a reliable gene knockout method for *E. gracilis*. In addition, because Cas12a‐based genome editing introduces long deletions at a single target site, genotyping can be simplified and performed in a high‐throughput manner using a high‐resolution electrophoresis system.

In the base‐editing experiments of *EgGSL2* using the LbCas12a RNP complex and ssODN, we observed a clear low paramylon phenotype in mutant *E. gracilis* cells (Figure 6), suggesting that amino acid residues C121 and/or W122 in the predicted N‐terminal glycoside hydrolase catalytic domain (Tanaka et al., 2017) is required for EgGSL2 function as a paramylon synthase. Base‐editing approaches can be used to identify enzyme active sites and post‐translational modification sites (such as phosphorylation), which can be a useful tool for basic research on gene function in *E. gracilis*. Furthermore, if should also be possible to improve enzyme activity by base editing, which would contribute to the establishment of useful metabolite‐producing strains through metabolic modification.

Through exploring the complex genomic landscape of *E. gracilis*, our study serves to both clarify its genetic intricacies and practical applications. Despite efforts in genome sequencing, the current state of the *E. gracilis* nuclear genome remains fragmented (Ebenezer et al., 2019), which may suggest the complexity of its genomic structure, likely due to a high prevalence of repetitive elements, closely related sequences and the possibility of segmental or complete genome duplication. Evidence for gene duplication at the individual gene level comes from our genome editing experiments, where we observed multiple variations at the edited sites in isolated pure strains. Building upon the successful application of genome editing technologies in polyploid crops (Zaman et al., 2019), as we also have demonstrated in *E. gracilis* (Ishikawa et al., 2022; Tamaki et al., 2023), genome editing proves useful for simultaneously introducing mutations into duplicated genes, thereby achieving complete knockout effects and directly facilitating trait modification. Looking ahead, forthcoming improvements in genome information will allow for more precise adjustments to editing sites, enabling individual modification of duplicated genes and the introduction of allele‐specific mutations. These advancements offer avenues for investigating the functional differentiation between duplicated genes and making precise regulation to molecular networks. In this study, we demonstrate our technique for site‐directed amino acid substitution, providing a toolset for precise alterations in enzyme functions, DNA‐protein interactions and interaction of protein multimers, which stand to advance our understanding and control over the complex genetic codes in *E. gracilis*.

Cas12a systems other than LbCas12a are also considered to be functional in *E. gracilis*. However, the general incubation temperature for this species is 25–28°C, suggesting that AsCas12a, whose activity is reported to decrease at temperatures below 37°C, may not be suitable for *E. gracilis* (Malzahn et al., 2019; Moreno‐Mateos et al., 2017). MAD7 (ErCas12a) is a Cas12a family nuclease isolated from *Eubacterium rectale*. It has low sequence homology (31%) with canonical Cas12a nucleases; as a royalty‐free nuclease, MAD7 is available to the public for academic and commercial use (Lin et al., 2021; Liu et al., 2020; Wierson et al., 2019). In plants, MAD7 editing efficiency is comparable to that of LbCas12a (Lin et al., 2021), suggesting that MAD7 may have potential for use in *E. gracilis*.

Compared with Cas9‐based genome editing, the frequency of cognate PAM sequences in Cas12a‐based genome editing is relatively low. To solve this problem, researchers have attempted to engineer Cas12a to broaden its PAM sequence recognition. For example, it has been reported that G532R/K595R (RR), G532R/K538V/Y542R (RVR) and G532R/K538V/Y542R/K595R (RVRR) engineered variants of LbCas12a can change its PAM specificity to TYYV, TWTV and TNTN, respectively (Gao et al., 2017; Tóth et al., 2018, 2020). It is expected that future introduction of these engineered Cas12a nucleases in *Euglena* will further expand its genome editing possibilities.

We demonstrated that our high‐efficiency genome editing method for *E. gracilis* using Cas12a‐based RNP complexes expands the toolbox for euglenoid genetic research. For example, genome editing using Cas12a in combination with Cas9 would enable more flexibility in designing target sequences, making it more feasible to generate amino acid substitutions in specific motifs or knock‐in tag sequences to characterize target proteins, which are currently restricted by target sequence design sites. Use of Cas12a would also make it possible to target and modify AT‐rich genomic regions such as promoters and intron sequences, which was difficult with Cas9. These new genetic tools are expected to accelerate engineering not only for basic research but also for the production of biomaterials using *Euglena*.

## AUTHOR CONTRIBUTIONS

**Toshihisa Nomura:** Conceptualization (lead); funding acquisition (equal); investigation (lead); methodology (lead); writing – original draft (lead); writing – review and editing (equal). **June‐Silk Kim:** Formal analysis (equal); investigation (supporting). **Marumi Ishikawa:** Investigation (supporting); writing – review and editing (supporting). **Kengo Suzuki:** Funding acquisition (equal); supervision (equal); writing – review and editing (supporting). **Keiichi Mochida:** Funding acquisition (equal); supervision (equal); writing – original draft (supporting); writing – review and editing (equal).

## CONFLICT OF INTEREST STATEMENT

This study was partially supported by a matching fund‐based research program between RIKEN and Euglena Co., Ltd.

## Supporting information


Appendix S1.

## Data Availability

The data sets that support the results of this study are available from the corresponding author upon reasonable request.
