# Supplementary material for: High‐efficiency genome editing by Cas12a ribonucleoprotein complex in Euglena gracilis
Source: Microb Biotechnol. 2024 Feb 8;17(2):e14393. doi: 10.1111/1751-7915.14393 (PMC10884871; doi:10.1111/1751-7915.14393)
Supplement: Supplementary file 1 — Appendix S1. [file MBT2-17-e14393-s001.docx]

**Supplementary figures**

# High-efficiency genome editing by Cas12a ribonucleoprotein complex in *Euglena gracilis*

Toshihisa Nomura*^1,2,3^, June-Silk Kim^1,4^, Marumi Ishikawa^2,5^, Kengo Suzuki^2,5^, Keiichi Mochida*^1,2,6,7,8^

**Author affiliations:**

^1^RIKEN Center for Sustainable Resource Science, 1-7-22 Suehiro-cho, Tsurumi-ku, Yokohama 230-0045, Japan

^2^RIKEN Baton Zone Program, 1-7-22 Suehiro-cho, Tsurumi-ku, Yokohama 230-0045, Japan

^3^Faculty of Agriculture, Yamagata University, Tsuruoka, Yamagata 997-8555, Japan

^4^Institute of Plant Science and Resources, Okayama University, Okayama, 710-0046, Japan

^5^euglena Co., Ltd., 5-33-1 Shiba, Minato-ku, Tokyo 108-0014, Japan

^6^Kihara Institute for Biological Research, Yokohama City University, 641-12 Maioka-cho, Totsuka-ku, Yokohama, Kanagawa 244-0813, Japan

^7^Graduate School of Nanobioscience, Yokohama City University, 1-7-29 Suehiro-cho, Tsurumi-ku, Yokohama, Kanagawa 230-0045, Japan
^8^School of Information and Data Sciences, Nagasaki University, Nagasaki, 852-8521, Japan

**Corresponding authors:** [keiichi.mochida@riken.jp](mailto:keiichi.mochida@riken.jp), [toshihisa.nomura@riken.jp](mailto:toshihisa.nomura@riken.jp)

**
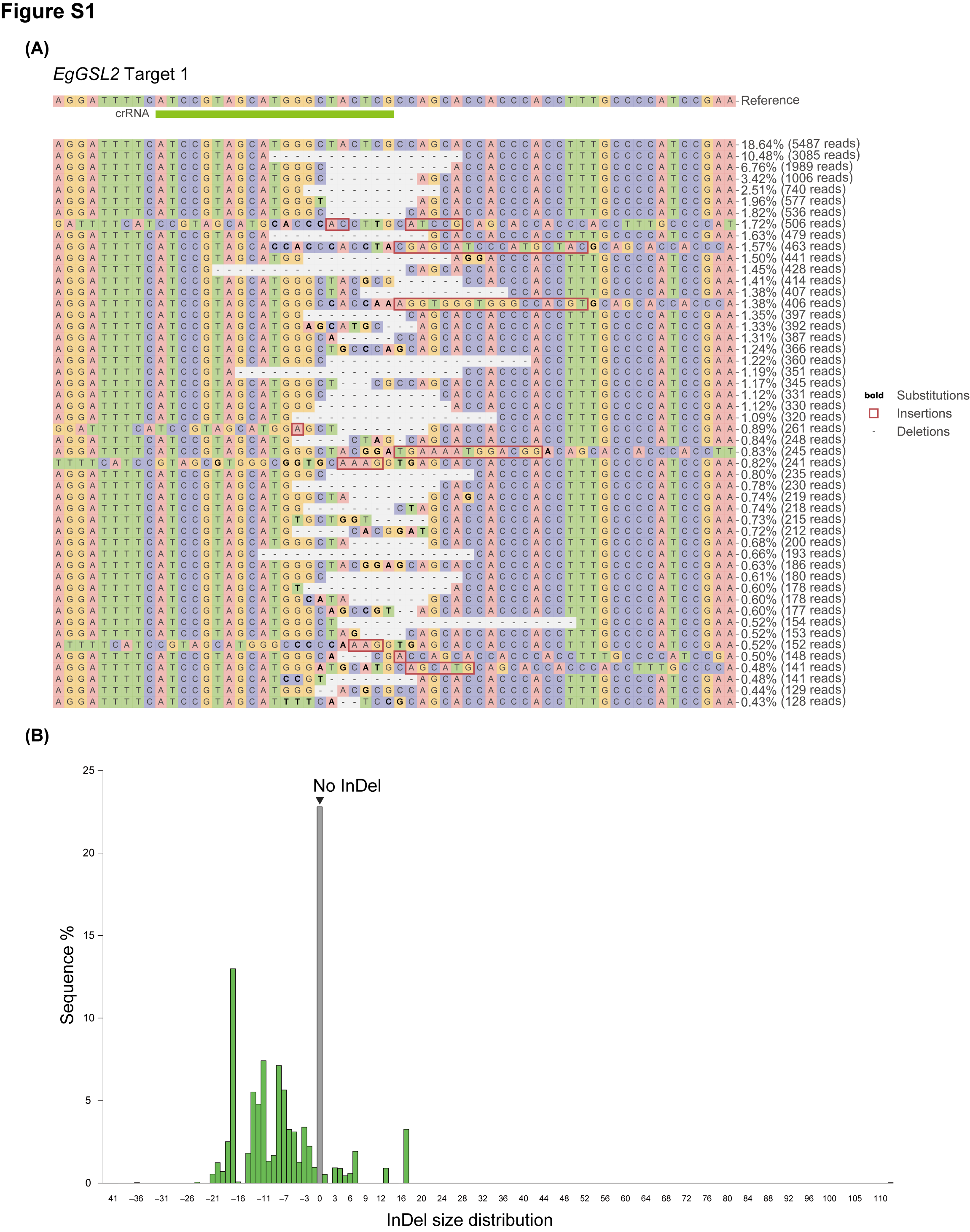
**

**FIGURE S1. Mutagenesis pattern of *EgGSL2* target site 1.** (A) Allele frequency table for *EgGSL2* target site 1 at 96 h after introduction of *EgGSL2*-targeting LbCas12a RNP complexes. (B) InDel size distribution for *EgGSL2* target site 1 at 96 h after introduction of *EgGSL2*-targeting LbCas12a RNP complexes.

**
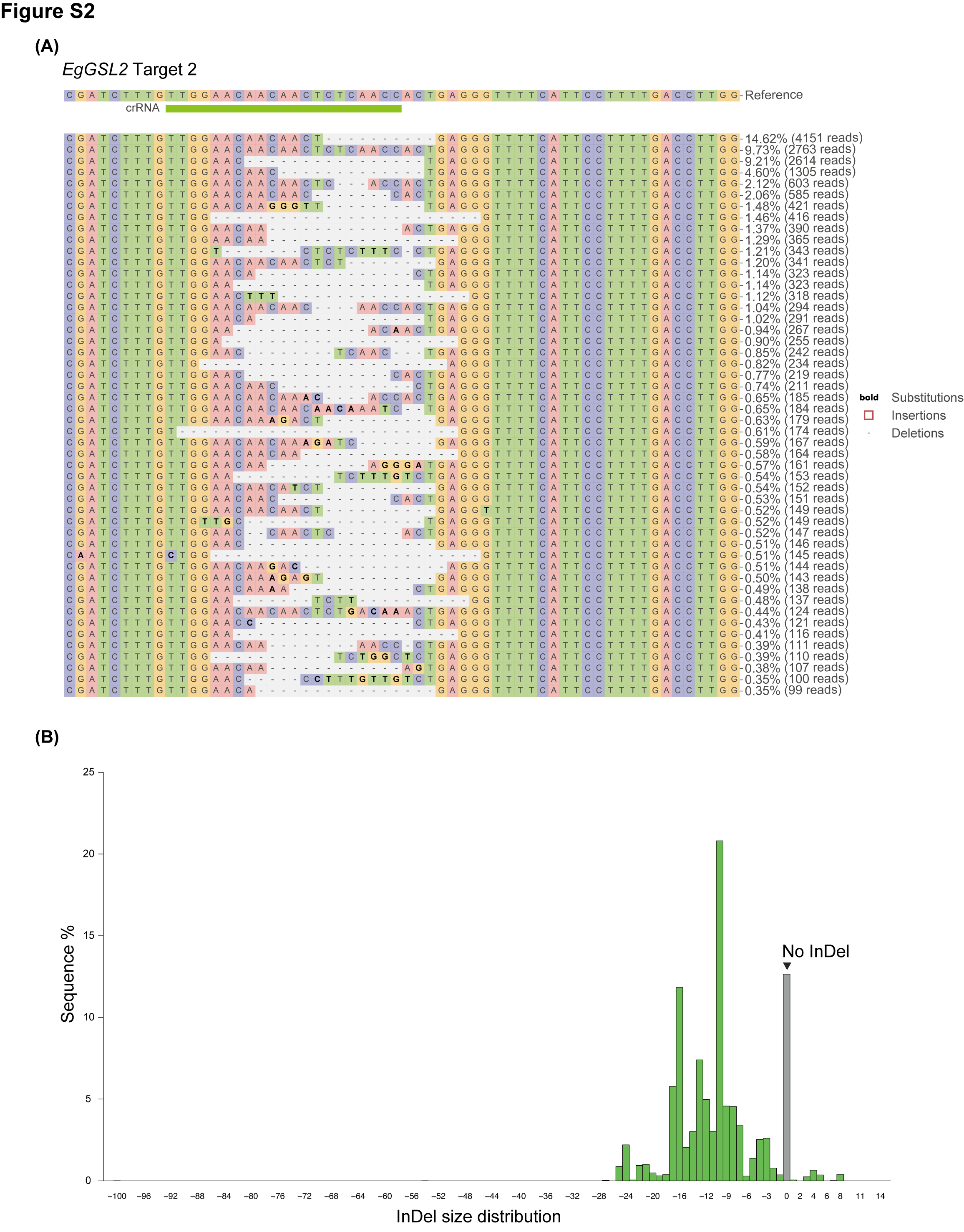
**

**FIGURE S2. Mutagenesis pattern of *EgGSL2* target site 2.** (A) Allele frequency table for *EgGSL2* target site 2 at 96 h after introduction of *EgGSL2*-targeting LbCas12a RNP complexes. (B) InDel size distribution for *EgGSL2* target site 2 at 96 h after introduction of *EgGSL2*-targeting LbCas12a RNP complexes.

**
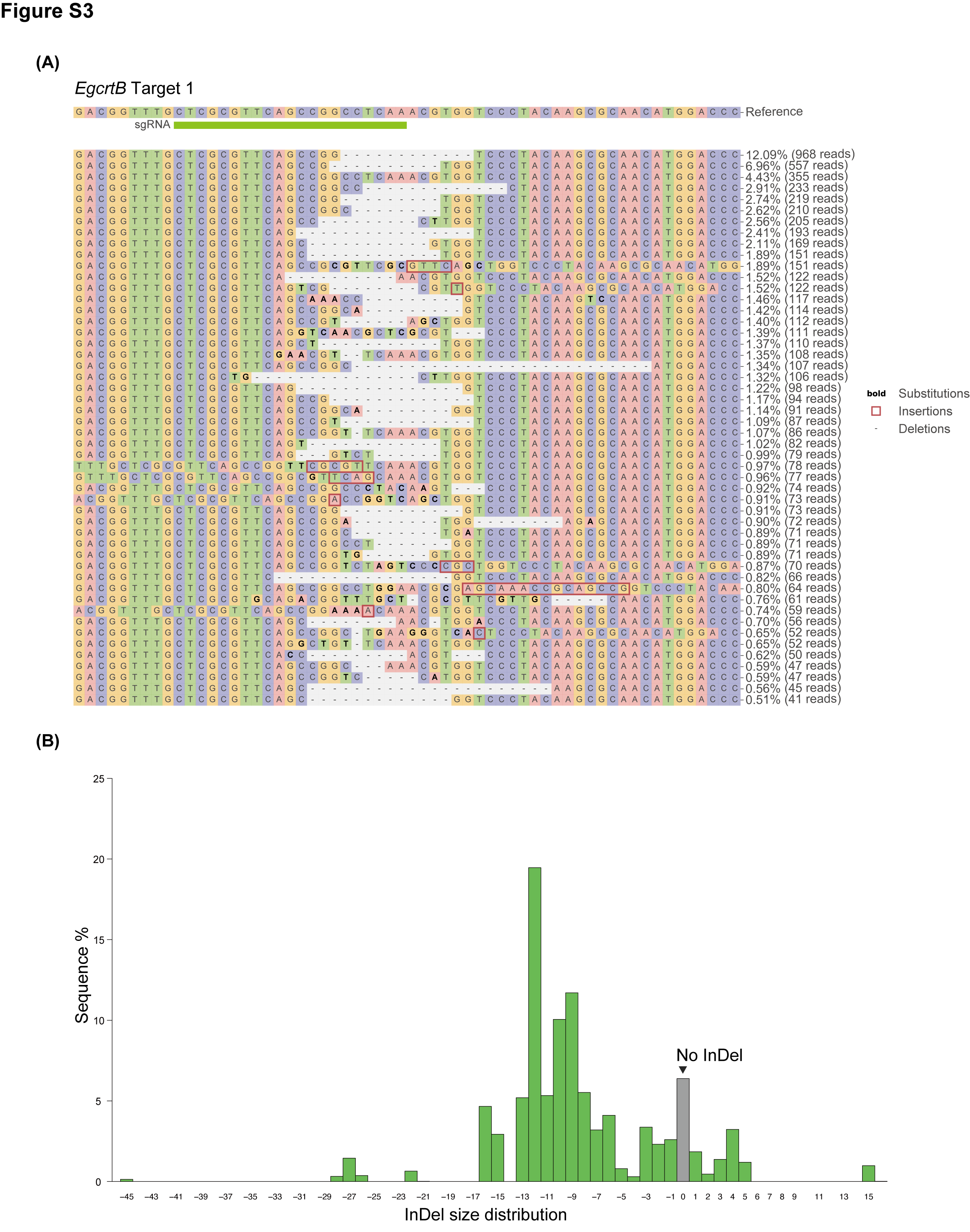
**

**FIGURE S3. Mutagenesis pattern of the *EgcrtB* target site 1.** (A) Allele frequency table for *EgcrtB* target site 1 at 96 h after introduction of *EgcrtB*-targeting LbCas12a RNP complexes. (B) InDel size distribution for *EgcrtB* target site 1 at 96 h after introduction of *EgcrtB*-targeting LbCas12a RNP complexes.

**
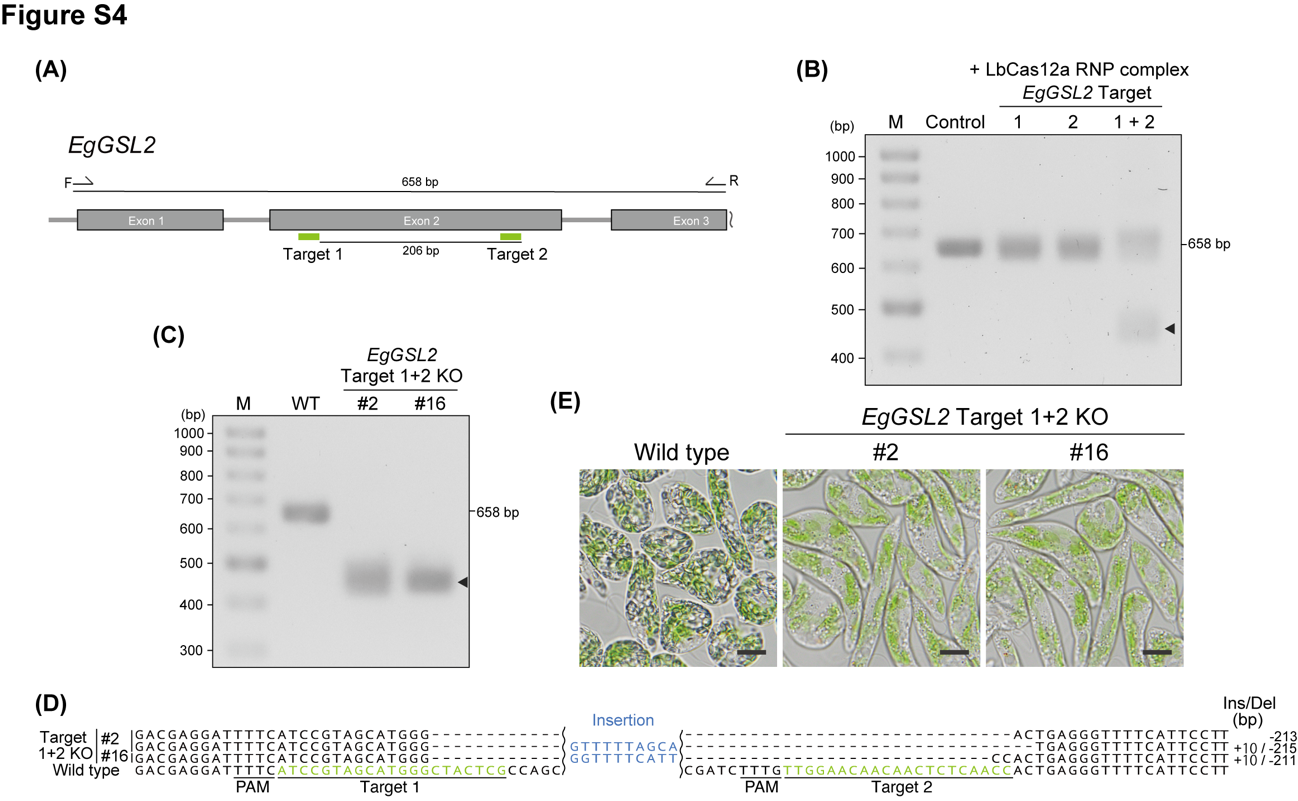
**

**FIGURE S4. Introduction of long deletions using two Cas12a RNP** **complexes.** (A) Diagram of the *EgGSL2* gene model and PCR product containing target sequences 1 and 2. (B) PCR products derived from the cell population 96 h after introduction of LbCas12a RNP complexes in the non-treated condition (Control) and two sets of LbCas12a RNP complexes targeting *EgGSL2*. Arrowheads indicate truncated PCR products. (C) PCR products derived from the wild-type and *EgGSL2* genome-edited strains. Arrowheads indicate truncated PCR products. (D) Alignment of genomic DNA sequences flanking the target sites in wild-type and *EgGSL2* genome-edited strains. (E) Representative images of wild-type and *EgGSL2* genome-edited strains after 2 days of growth in KH medium. Scale bar, 10 μm.

**TABLE S1. DNA oligos used in this study.**

|  |  | Number of checked strains | Chlorosis strains | Chlorosis strain % |
| --- | --- | --- | --- | --- |
| Control | Trial 1 | 29 | 0 | 0 |
|  | Trial 2 | 31 | 0 | 0 |
|  | Trial 3 | 24 | 0 | 0 |
|  | Trial 4 | 27 | 0 | 0 |
| + LbCas12a RNP complex  (*EgcrtB* target) | Trial 1 | 27 | 4 | 14.8 |
|  | Trial 2 | 26 | 8 | 30.8 |
|  | Trial 3 | 25 | 7 | 28.0 |
|  | Trial 4 | 26 | 6 | 23.1 |

**TABLE S2. Percentage of the strains with chlorosis phenotype in isolated strains.**

|  |  | Number of checked strains | Knock-in strains | Knock-in strain % |
| --- | --- | --- | --- | --- |
| Control | Trial 1 | 26 | 0 | 0 |
|  | Trial 2 | 31 | 0 | 0 |
|  | Trial 3 | 24 | 0 | 0 |
|  | Trial 4 | 24 | 0 | 0 |
| + LbCas12a RNP complex  + ssODNs  (Knock-in) | Trial 1 | 22 | 8 | 36.4 |
|  | Trial 2 | 21 | 9 | 42.9 |
|  | Trial 3 | 23 | 10 | 43.5 |
|  | Trial 4 | 20 | 5 | 25.0 |

**TABLE S3. Percentage of the strains with precise knock-in sequence in isolated strains.**

|  |  | Number of checked strains | Base editing strains | Base editing strain % |
| --- | --- | --- | --- | --- |
| Control | Trial 1 | 24 | 0 | 0 |
|  | Trial 2 | 24 | 0 | 0 |
|  | Trial 3 | 24 | 0 | 0 |
|  | Trial 4 | 24 | 0 | 0 |
| LbCas12a RNP complex ssODNs | Trial 1 | 23 | 13 | 56.5 |
|  | Trial 2 | 27 | 8 | 29.6 |
|  | Trial 3 | 17 | 6 | 35.3 |
|  | Trial 4 | 15 | 6 | 40.0 |

**TABLE S4. Percentage of the strains with precise base editing sequence in isolated strains.**
